# Histology of Cardiac Sarcoidosis with Novel Considerations Arranged upon a Pathologic Basis

**DOI:** 10.3390/jcm11010251

**Published:** 2022-01-04

**Authors:** Shu Kato, Yasuhiro Sakai, Asako Okabe, Yoshiaki Kawashima, Kazuhiko Kuwahara, Kazuya Shiogama, Masato Abe, Hiroyasu Ito, Shin’ichiro Morimoto

**Affiliations:** 1 Postgraduate Clinical Training Center, Fujita Health University Hospital, Aichi 470-1192, Japan; shu.kato@fujita-hu.ac.jp; 2 Department of Joint Research Laboratory of Clinical Medicine, Fujita Health University School of Medicine, Aichi 470-1192, Japan; hiroyasu.ito@fujita-hu.ac.jp; 3 Department of Diagnostic Pathology, Kansai Medical University Hospital, Osaka 573-1191, Japan; milleq@fujita-hu.ac.jp; 4 Department of Pathology, Fujita Health University Bantane Hospital, Aichi 454-8509, Japan; ykawa@fujita-hu.ac.jp; 5 Department of Diagnostic Pathology, Fujita Health University School of Medicine, Aichi 470-1192, Japan; kazukuwa@fujita-hu.ac.jp; 6 Department of Morphology and Pathological Diagnosis, Fujita Health University School of Medical Sciences, Aichi 470-1192, Japan; kazshio@fujita-hu.ac.jp (K.S.); masabe@fujita-hu.ac.jp (M.A.); 7 Department of Cardiology, Fujita Health University School of Medicine, Aichi 470-1192, Japan; morimoto@fujita-hu.ac.jp

**Keywords:** cardiac sarcoidosis, histiocytic myocarditis, multinucleated giant cells, *Cutibacterium (Propionibacterium) acnes*, pro-inflammatory (M1) macrophage, microgranuloma, CD4/CD8 T-cell ratio, lymphangiogenesis, confluent fibrosis, fatty infiltration

## Abstract

Sarcoidosis is a rare disease of isolated or diffuse granulomatous inflammation. Although any organs can be affected by sarcoidosis, cardiac sarcoidosis is a fatal disorder, and it is crucial to accurately diagnose it to prevent sudden death due to dysrhythmia. Although endomyocardial biopsy is invasive and has limited sensitivity for identifying granulomas, it is the only modality that yields a definitive diagnosis of cardiac sarcoidosis. It is imperative to develop novel pathological approaches for the precise diagnosis of cardiac sarcoidosis. Here, we aimed to discuss commonly used diagnostic criteria for cardiac sarcoidosis and to summarize useful and novel histopathologic criteria of cardiac sarcoidosis. While classical histologic observations including noncaseating granulomas and multinucleated giant cells (typically Langhans type) are the most important findings, others such as microgranulomas, CD68^+^ CD163^−^ pro-inflammatory (M1) macrophage accumulation, CD4/CD8 T-cell ratio, *Cutibacterium acnes* components, lymphangiogenesis, confluent fibrosis, and fatty infiltration may help to improve the sensitivity of endomyocardial biopsy for detecting cardiac sarcoidosis. These novel histologic findings are based on the pathology of cardiac sarcoidosis. We also discussed the principal histologic differential diagnoses of cardiac sarcoidosis, such as tuberculosis myocarditis, fungal myocarditis, giant cell myocarditis, and dilated cardiomyopathy.

## 1. Introduction

Sarcoidosis is a systemic inflammatory disease of unknown etiology and is characterized by noncaseating granulomas that may involve any organs of the body, particularly the lungs, lymph nodes, skin, liver, spleen, and eyes [1]. Cardiac sarcoidosis is rare and is clinically recognized in approximately 2% to 7% of patients with sarcoidosis [2]. It is categorized into two types: isolated cardiac sarcoidosis, accounting for 23% to 54% of cardiac sarcoidosis, and systemic sarcoidosis with cardiac involvement [3,4]. In cardiac sarcoidosis, cardiac symptoms depend on the location and extent of sarcoidal granulomas in the heart, and cardiac involvement occasionally manifests in severe dysrhythmias, valvular dysfunction, heart failure, and sudden cardiac death when sarcoidal granulomas destroy cardiomyocytes, valves, and the impulse conducting system [3,5].

Cardiac sarcoidosis typically occurs in the 20s to 60s age group and is more common in women than in men [5,6]. The incidence and severity of cardiac sarcoidosis notably differs across ethnic and racial groups. The Japanese have a higher incidence of cardiac sarcoidosis compared to Caucasians and African-Americans, and cardiac sarcoidosis is the leading cause of death (77% to 85%) from sarcoidosis in Japan, whereas only 13% to 50% of sarcoidosis-related deaths are due to cardiac sarcoidosis in the United States [2,7,8,9,10].

Cardiac sarcoidosis is discovered at autopsy in 15% to 90% of cases of systemic sarcoidosis, and it can affect anywhere in the heart [4,5,6,7,8,9,10]. However, the most common location affected by sarcoidal granulomas is the left ventricular free wall, followed by the basal interventricular septum and right ventricular and atrial walls [8]. Sarcoidal granulomas spread from the subepicardial myocardium to the epicardium and pericardium and may be associated with exudative pericardial effusions. Affected lesions in the heart are grossly sprinkled, irregularly shaped, whitish “eraser-like” masses with rubbery hardness [11].

It is often difficult to recognize cardiac sarcoidosis via antemortem endomyocardial biopsies. It is typically diagnosed when endomyocardial biopsy demonstrates noncaseating granulomas; however, endomyocardial biopsy has low sensitivity (approximately 20% to 30%) for the diagnosis owing to the small size and high sampling error rate associated with biopsy specimens [12,13]. In addition, specimens are usually obtained from the right ventricular septum because it is safely accessible by traditional transvenous cardiac biopsy, although sarcoidal granulomas appear more commonly in the left ventricular wall and basal interventricular septum, which are regions that are difficult to biopsy [7]. Electrophysiological or image-guided biopsy procedures have been reported to be useful for identifying low voltage areas, which may correspond to granulomas and may be targeted for biopsy, resulting in an increased diagnostic rate [14,15]. Alongside these novel procedures, it is also imperative to develop novel pathological approaches for the precise diagnosis of cardiac sarcoidosis.

Here, we introduce commonly used diagnostic criteria for cardiac sarcoidosis and discuss the significance of endomyocardial biopsy. Thereafter, we summarize useful and novel histopathologic criteria of cardiac sarcoidosis. Finally, we discuss the principal differential diagnoses of cardiac sarcoidosis.

## 2. Diagnostic Criteria for Cardiac Sarcoidosis

**Figure 1:**
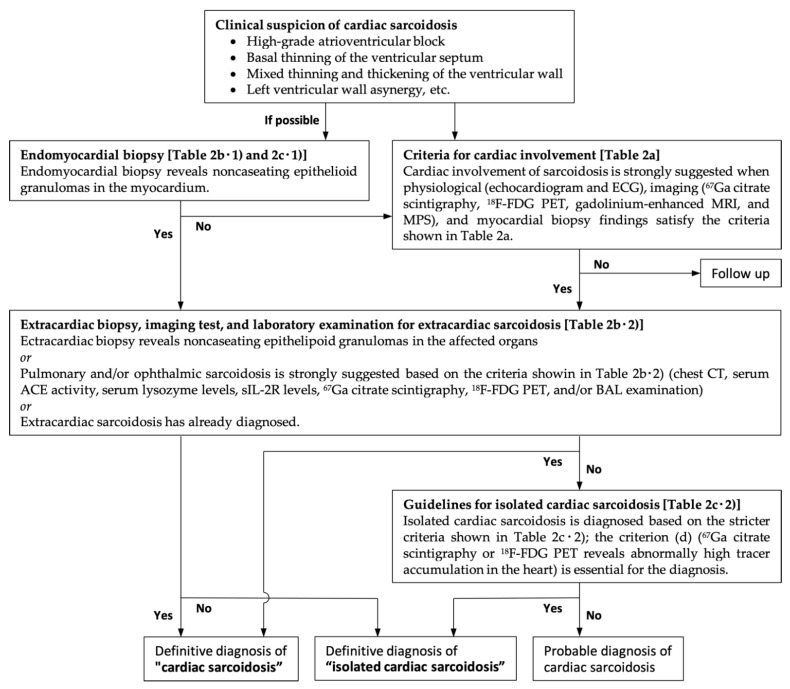
The diagnostic algorism for cardiac sarcoidosis based on the revised Japanese Ministry of Health and Welfare criteria. ECG, electrocardiogram; ^67^Ga, gallium-67; ^18^F-FDG PET, fluorine-18 fluorodeoxyglucose positron emission tomography; MRI, magnetic resonance imaging, MPS, myocardial perfusion scintigraphy; CT, computed tomography; ACE, angiotensin converting enzyme; sIL-2R, soluble interleukin 2 receptor, BAL, bronchoalveolar lavage.

The most commonly used diagnostic criteria for cardiac sarcoidosis are the 2014 Heart Rhythm Society Expert Consensus Statement on the Diagnosis and Management of Arrhythmias Associated with Cardiac Sarcoidosis (see Table 1) [16] and the revised Japanese Ministry of Health and Welfare criteria (see Table 2 and Figure 1) [4,17]. In both criteria, cardiac sarcoidosis can be definitively diagnosed based on the presence of extracardiac sarcoidosis in conjunction with other clinical, laboratory, and radiological findings that suggest cardiac involvement. Endomyocardial biopsy is recommended in the patients who lack histologic confirmation of extracardiac sarcoidal granulomas (in cases with isolated cardiac sarcoidosis or systemic sarcoidosis without apparent endomyocardial biopsy findings). However, the sensitivity is low owing to sampling errors that may occur due to the patchy distribution of the disease.

## 3. Histologic Findings Arranged upon a Pathologic Basis

Most pathologists diagnose cardiac sarcoidosis depending on the presence of sarcoidal granulomas. However, the diagnostic sensitivity of endomyocardial biopsy is low because sarcoidal granulomas are rarely detected in many cases. Here, we emphasize the histologic findings of cardiac sarcoidosis based on the temporal evolution and significance of the morphologic alterations.

### 3.1. Interstitial Phase of Cardiac Sarcoidosis (Histiocytic Myocarditis)

Although the antigenic cause of sarcoidosis has not been elucidated, most pathologists believe that sarcoidosis is caused by an antigen-driven immune response and that the initial trigger involves antigen-presenting cells such as dendritic cells and macrophages presenting the unknown antigen to lymphocytes. Accordingly, CD4^+^ helper T cells are predominantly recruited, expanded oligoclonally, activated, and polarized into the T helper 1 (Th1) phenotype, which results in a CD4/CD8 T-cell ratio of approximately 10:1 [18,19,20,21]. Thereafter, lymphocyte infiltration, interstitial edema, and subsequent accumulation of CD209^+^ dendritic cells and CD68^+^ macrophages are caused by T-cell-derived Th1 cytokines such as interleukin (IL)-2 and interferon (IFN)-γ, which progress into histiocytic myocarditis. Most accumulated macrophages are CD68^+^ CD163^−^ pro-inflammatory macrophages (M1 macrophages), whereas CD68^+^ CD163^+^ anti-inflammatory macrophages (M2 macrophages) are less often observed [22,23].

**Figure 2:**
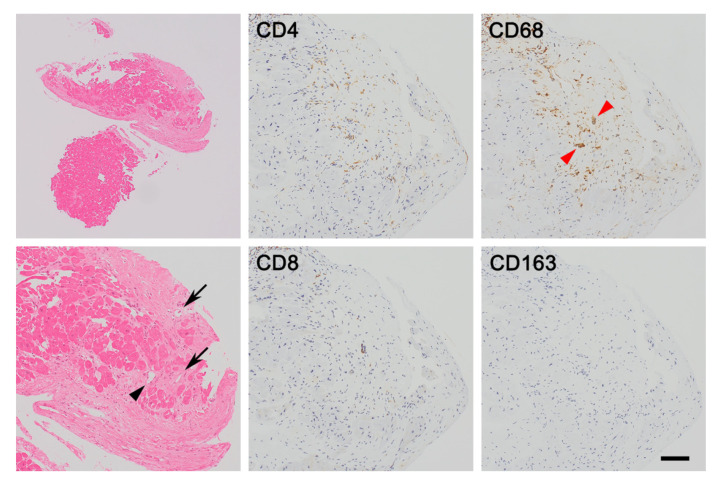
Interstitial phase of cardiac sarcoidosis (histiocytic myocarditis). Endomyocardial biopsy shows that macrophages accumulate sparsely as well as a few lymphocytes infiltrating in the lesions where cardiomyocytes have disappeared. As seen in this case, it is difficult to diagnose interstitial phase of cardiac sarcoidosis because apparent sarcoidal granulomas are seldom observed. However, the morphological findings, such as lymphangiogenesis (arrows), fatty infiltration (black arrowhead), and microgranulomas (red arrowheads), and the immunohistochemical results including high CD4/CD8 T-cell ratio and CD68^+^ CD163^−^ M1 macrophage phenotype are suggestive of cardiac sarcoidosis. Bar = 250 μm for a lower magnification field, 100 μm for higher magnification fields.

In this phase, granuloma formation is not obvious, and pathologists can hardly diagnose cardiac sarcoidosis from endomyocardial biopsy specimens (Figure 2). A recent report indicates that an increased number of dendritic cells and reduced number of M2 macrophages in the myocardium in biopsy specimens can serve as histologic surrogate findings for the diagnosis even if sarcoidal granulomas are not clearly observed in the myocardium [22].

Thereafter, the M1 macrophages activated by contact interaction with CD4^+^ helper T cells via CD40 and the subsequent secretion of IFN-γ turn into epithelioid cells that have an elongated shape, a pale and eosinophilic finely granular cytoplasm, and a central ovoid nucleus [19,20,21]. Some epithelioid cells fuse to form multinucleated giant cells. These are typically Langhans giant cells, in which multiple nuclei are arranged linearly toward the cell periphery in a horseshoe-like semicircular shape; foreign body giant cells and Turton giant cells can also be observed. Accordingly, activated M1 macrophages, multinucleated giant cells, and dendritic cells occasionally form ill-defined granulomas [22,23].

As M1 macrophages accumulate with CD4^+^ helper T cell infiltration, further migration and activation of lymphocytes and macrophages supplied from the right paratracheal lymph nodes are retrograded owing to lymphatic drainage obstruction [24]. As a result, lymphangiogenesis is often induced, and the number of D2-40^+^ lymph vessels is increased in the affected lesions [25]. The basal interventricular septum, right-sided interventricular septum, lateral left ventricular wall, and anterolateral papillary muscle, where lymph fluid is drained from the right paratracheal lymph nodes, have an extensive lymphatic capillary network, and may be the most frequently affected areas in cardiac sarcoidosis [24].

### 3.2. Granulomatous Phase of Cardiac Sarcoidosis

**Figure 3:**
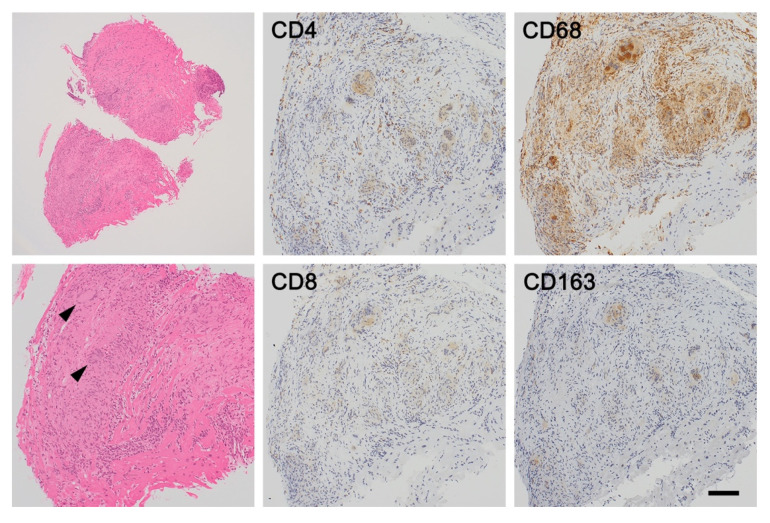
Granulomatous phase of cardiac sarcoidosis. Well-formed noncaseating granulomas (arrowhead) are composed of aggregates of tightly clustered epithelioid macrophages, often with multinucleated giant cells of Langhans and foreign-body types. Few CD4^+^ helper T cells are surrounded by granulomas, and they are often described as “naked granulomas”. Most of macrophages and giant cells accumulated express CD68^+^ CD163^−^ M1 phenotype. Bar = 250 μm for a lower magnification field, 100 μm for higher magnification fields.

As the disease progresses, several cytokines in the local environment including IL-8, tumor necrosis factor-α, and macrophage inflammatory protein 1α recruit additional T cells and macrophages [26,27,28]. This contributes to the formation of tighter granulomas in which activated M1 macrophages, multinucleated giant cells, and a few CD4^+^ helper T cells are centrally accumulated, surrounded by a small number of CD8^+^ cytotoxic T cells. Typically, these well-defined granulomas are morphologically “naked”, which means few lymphocytes and plasma cells are “worn” by granulomas (Figure 3). Most textbooks describe sarcoidal granulomas as “naked” and “noncaseating”, and these are the histologic hallmarks of sarcoidosis; however, the intensity of lymphocyte and plasma cell infiltration varies among patients, and “non-naked” epithelioid granulomas can also be observed. This means that cardiac sarcoidosis cannot be ruled out even when granulomas are not “naked”. In addition, necrosis may be present in up to 20% of cases (necrotizing sarcoid granulomatosis) [29].

Schaumann bodies, which are oval, concentric laminations of calcified proteins, are often identified in multinucleated giant cells in sarcoidal granulomas (up to 88% of cases), whereas asteroid bodies, which are star-shaped structures composed of filamentous microtubular materials, are less frequently observed [30]. These bodies of multinucleated giant cells in granulomas are nonspecific for sarcoidosis.

### 3.3. Fibrous Phase of Cardiac Sarcoidosis

Cytokine signature transitions from Th1 to Th2 predominance are probably due to a response to persistent granulomatous inflammation [31,32]. Th2 cells secrete IL-4, IL-5, and IL-13, which are responsible for the alternative pathway of macrophage activation, resulting in the induction of M2 macrophages [32,33]. Consequently, there may be more CD68^+^ CD163^+^ M2 macrophages in this phase than in the interstitial phase, although CD68^+^ CD163^−^ M1 macrophages are unalterably observed [31].

M2 macrophages potentially produce cytokine signals that stimulate fibrosis, such as transforming growth factor (TGF)-β and chemokine CC motif ligand (CCL) 18. TGF-β leads to the recruitment, activation, and proliferation of fibroblasts, and stimulates them to produce collagen, fibronectin, and proteoglycans [34]. TGF-β inhibits collagen degradation by deactivating matrix metalloproteinases and activating proteinase inhibitors. CCL18 also directly induces fibroblasts to produce collagen [35]. Therefore, lesions where more than 30 to 50 cardiomyocytes culminate in necrosis are replaced with confluent fibrosis. Lipocytes occasionally infiltrate the myocardial complementary spaces where the volume of the myocardium is decreased owing to confluent fibrosis; this phenomenon is called fatty infiltration [36].

**Figure 4:**
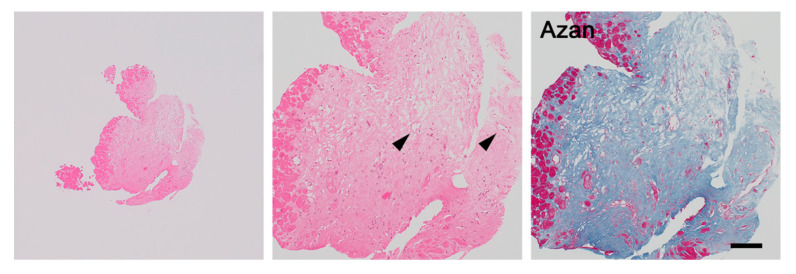
Fibrous phase of cardiac sarcoidosis. Although it is quite difficult to diagnose cardiac sarcoidosis in endomyocardial biopsy, confluent fibrosis and lymphangiogenesis (arrowheads) may be helpful for diagnosing cardiac sarcoidosis. Bar = 500 μm for a lower magnification field, 100 μm for higher magnification fields.

With further progression, sarcoidal granulomas develop fibrotic changes in some but not all patients with severe or long-standing sarcoidal inflammation. Fibrotic alterations are stimulated by several cytokines such as TGF-β, IL-5, and IL-7, which may be induced by macrophage phenotype switching from M1 to M2 and T-cell transition from Th1 to Th2 transition [37,38]. Several reports indicate that TGF-β2 and TGF-β3 SNPs are associated with fibrotic sarcoidosis [39,40]. The changes usually begin at the periphery of the granulomas and, eventually, the granulomas are totally replaced by dense confluent fibrosis [8,10,41]. When tissues are sampled from these areas, viable granulomas are not recognized; this is one of the causes of the difficulty to diagnose cardiac sarcoidosis in endomyocardial biopsy (Figure 4). Finally, there can be progression to dilated cardiomyopathy and ventricular aneurysm.

## 4. Novel Surrogate Histologic Findings

A typical histopathologic feature of cardiac sarcoidosis is noncaseating granuloma formation similar to that observed in extracardiac sarcoidosis. As described above, however, noncaseating granulomas are seldom observed in endomyocardial biopsy specimens, particularly in the interstitial phase of cardiac sarcoidosis; therefore, diagnostic confirmation for cardiac sarcoidosis is often difficult. Several reports indicate that a combination of other novel surrogate histologic findings, such as microgranulomas, M1 macrophage accumulation, lymphangiogenesis, *Cutibacterium acnes* components, confluent fibrosis, and fatty infiltration, may be useful for the histological diagnosis.

### 4.1. Microgranulomas

Granulomas are usually defined as collections of activated macrophages surrounded by a collar of lymphocytes, particularly T lymphocytes. Activated macrophages, also called epithelioid cells, have an eosinophilic granular cytoplasm with an indistinct cell boundary. In histiocytic myocarditis, although typical sarcoidal granulomas are rarely distinctly recognized, several activated mononucleated macrophages collected in minute ill-defined nodules are occasionally termed “microgranulomas”. [36] There is no consensus on the size of the microgranuloma; however, it is representatively a minute nodular collection of 5 to 10 macrophages. Microgranulomas may be a diagnostic basis for cardiac sarcoidosis [36].

### 4.2. CD68^+^ CD163^−^ M1 Macrophage Accumulation

Typical myocarditis, including lymphocytic myocarditis and hypersensitivity (eosinophilic) myocarditis, is characterized by an interstitial inflammatory cell infiltrate composed of lymphocytes and eosinophils. However, in cardiac sarcoidosis, the number of macrophages accumulated is approximately increased three or more times than normal. Giant cell myocarditis also shows interstitial accumulation of macrophages and multinucleated giant cells. Honda et al. reported that the CD163/CD68 macrophage ratio (≤0.70) demonstrated high sensitivity (81.4%) and specificity (84.0%), and, furthermore, the combination of the CD163/CD68 macrophage ratio (≤0.70) and CD209^+^ dendritic cell density (≥13/0.1 mm^2^) demonstrated much higher specificity (100%) for cardiac sarcoidosis [22]. Because sarcoidosis is notably associated with Th1/Th17 multisystem abnormity, an increased number of CD68^+^ CD163^−^ M1 macrophages and CD209^+^ dendritic cells and a decreased number of CD68^+^ CD163^+^ M2 macrophages may be a histologic surrogate for the diagnosis of cardiac sarcoidosis [22,42].

### 4.3. Lymphangiogenesis

A recent study revealed the relationship between sarcoidal granulomas and lymphatic network and lymph vessel proliferation in cardiac sarcoidosis, as previously suggested in pulmonary sarcoidosis [24,25]. D2-40 antibodies recognizing podoplanin can detect lymphatic endothelium, and D2-40^+^ lymph vessel proliferation can be helpful for distinguishing between sarcoidal and other chronic/fibrous inflammations in the heart.

Oe et al. reported that the number of lymphatic vessels was significantly increased, not only in histologically diagnosed cardiac sarcoidosis but also in clinically diagnosed cardiac sarcoidosis that lacked apparent endomyocardial biopsy findings [24]. Because lymphatic drainage may increase in tissues with granuloma formation and induce lymphangiogenesis, an increased number of lymph vessels may serve as a good surrogate marker for the diagnosis of cardiac sarcoidosis [24].

### 4.4. Cutibacterium Acnes Components

Although the cause of sarcoidosis remains undetermined, an infectious origin has long been suspected. One of the candidates is tuberculous and nontuberculous *Mycobacterium* spp.; in several studies, mycobacterial DNA similar to those of *M. tuberculosis*, *M. avium, M. kansasii*, and *M. marinum* was detected in approximately one to two-thirds of sarcoidosis cases, although bacterial bodies of *Mycobacterium* spp. were not detected [1,8,43,44,45,46,47,48,49,50]. Another important candidate is *C. acnes* (formerly *Propionibacterium acnes*), which is the only microorganism to be isolated from sarcoidal lesions [51,52,53,54,55,56]. A previous study showed that *C. acnes* was isolated from lymph node biopsy samples in 78% of patients with sarcoidosis in Japan [56].

Recently, Negi et al. developed a monoclonal antibody that recognizes the cell membrane-bound lipoteichoic acid of *C. acnes* (also called anti-*P. acnes* antibody; PAB) [57]. Asakawa et al. indicated that *C. acnes* components were detected immunohistochemically using PAB in 63% of sarcoidal granulomas, 63% of massive (≥14 inflammatory cells) inflammatory foci, and 46% of minimal (<14 inflammatory cells) inflammatory foci in patients with cardiac sarcoidosis, whereas no reactivity was shown in granulomas and inflammatory foci in patients with non-sarcoidal myocarditis and cardiomyopathies [58]. Thus, *C. acnes* components can be detected not only in granulomas but also in histiocytic myocarditis without apparent granulomas. In addition, PAB-positive granular substances originating from *C. acnes* can also be identified by PAB. These results indicate that immunohistochemical analysis for *C. acnes* components is very useful for diagnosing cardiac sarcoidosis [58].

### 4.5. Confluent Fibrosis and Fatty Infiltration

Microgranulomas and/or histiocytic accumulation are always associated with confluent fibrosis and fatty infiltration [36]. Confluent fibrosis is edematous fibrous tissue where more than 30 to 50 necrotized cardiomyocytes are replaced. However, slender collagen fibers without cardiomyocyte injury and multifocal spotty fibrosis following individual cardiomyocyte damage are not considered confluent fibrosis. Masson trichrome and/or Azan staining are helpful for detecting confluent fibrosis. Fatty infiltration within loose connective tissue around granulomas is also a histologic finding that is often observed in sarcoidosis. However, it is occasionally difficult to distinguish between normal interstitial fatty tissue and fatty infiltration from the epicardium after myocardial inflammation. Fatty infiltration is often recognized as individual lipocytes in the myocardial scar, accumulated lipocytes in the subendocardium, and lipoblasts with small and bubbly cytoplasm.

The observation of both findings indicates probable sarcoidosis on endomyocardial biopsy [36]. Confluent fibrosis or fatty infiltration is considered weak pathological evidence and both are interpreted as nonspecific findings because they are also observed in myocardial infarction, cardiac hypertrophy, cardiomyopathy, and hypoxia.

## 5. Histologic Differential Diagnoses

Although endomyocardial biopsy can provide a definitive diagnosis of cardiac sarcoidosis, most histologic findings are nonspecific, as discussed above. Although the symptoms of cardiac sarcoidosis, such as ventricular arrhythmias, heart block, and failure, may be helpful for pathological diagnosis, myocarditis with lymphocyte infiltration and macrophage accumulation is seen not only in cardiac sarcoidosis but also in other heart diseases, including dilated cardiomyopathy, giant cell myocarditis, tuberculous myocarditis, and fungal myocarditis. The differential diagnoses are categorized into granulomatous disorders of infectious and noninfectious causes.

### 5.1. Infectious Causes (Tuberculosis and Fungal Infection)

Tuberculosis may present with similar clinical symptoms and histologic findings. Unlike cardiac sarcoidosis, tuberculous lesions first involve the pericardium and then spread to the myocardium. Tuberculoid granulomas are usually necrotizing, less cohesive than sarcoidal granulomas, and accompanied by a large rim of lymphocytes. Tuberculosis can be screened for when acid-fast bacilli are detected in and around granulomas by Ziehl–Neelsen staining.

Fungal myocarditis, which is generally a disseminated infection, basically occurs in immunosuppressed, immunocompromised, and/or neutropenic patients, and rarely affects patients with normal immunity. Grocott methenamine staining is valuable for distinguishing between fungal myocarditis and other granulomatous inflammations [8].

### 5.2. Giant Cell Myocarditis

**Figure 5:**
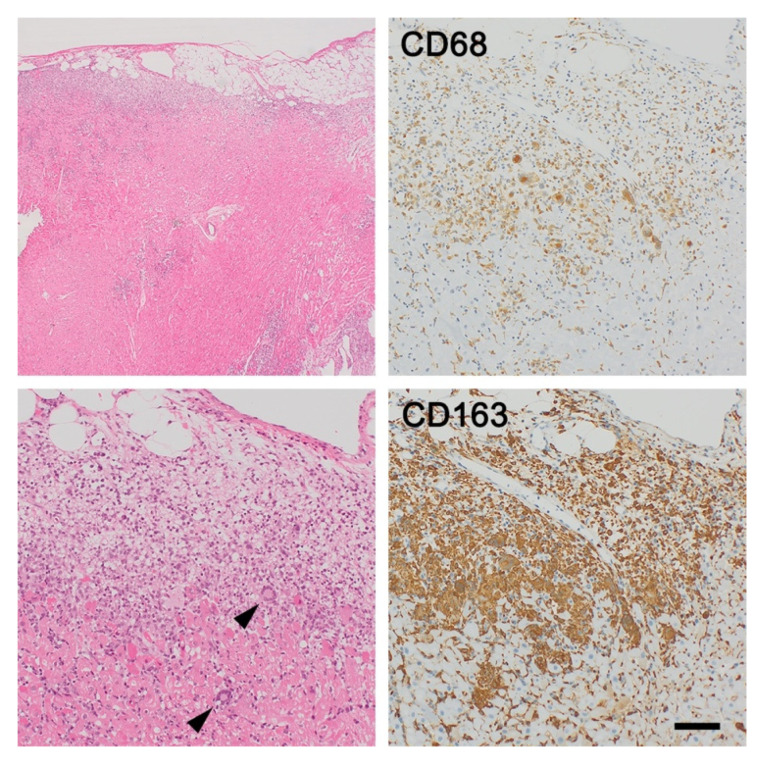
Giant cell myocarditis. Giant cell myocarditis and cardiac sarcoidosis are sometimes notably difficult to distinguish. In this case, subepicardial predominance is unusual for cardiac sarcoidosis, and not only macrophage accumulation but also lots of lymphocyte and eosinophil infiltrations are compatible with giant cell myocarditis. Immunohistochemistry is also valuable for pathological diagnosis; the number of CD68^+^ CD163^+^ M2 macrophages accumulated is greater compared with that of CD68^+^ CD163^−^ M1 macrophages, suggesting giant cell myocarditis rather than cardiac sarcoidosis. Bar = 500 μm for a lower magnification field, 100 μm for higher magnification fields.

Giant cell myocarditis is a rare, virus-negative, and usually fatal myocarditis that affects young and middle-aged adults and is attributed to T cell-mediated inflammation. Approximately 20% of cases are associated with systemic autoimmune disease. Histologic examination is essential in making a final diagnosis. Giant cell myocarditis is characterized by myocardial inflammation including lymphocytes, eosinophils, macrophages, and multinucleated giant cells as well as cardiomyocyte necrosis (Figure 5) [59].

Giant cell myocarditis and cardiac sarcoidosis are sometimes notably difficult to distinguish because they have many similar clinical manifestations, histopathological findings, and pathoetiology. Although there is some controversy regarding whether giant cell myocarditis and cardiac sarcoidosis are different phenotypes of a single disease or two distinct entities, some histological differences have been identified [60]. Granuloma formation and subsequent fibrosis are frequently observed and could serve as a hallmark of cardiac sarcoidosis, whereas no or few granulomas are observed in giant cell myocarditis [8,61]. However, prominent cardiomyocyte necrosis and different inflammatory cell infiltrates containing multinuclear giant cells and eosinophils histologically suggest giant cell myocarditis [62].

Moreover, some reports indicate that immunohistochemistry may be helpful for diagnosis; although both are caused by T cell-mediated autoimmune inflammation, CD4^+^ helper T cells are dominantly recruited in cardiac sarcoidosis as described above, whereas CD8^+^ cytotoxic T cells more often occur in giant cell myocarditis [30].

### 5.3. Dilated Cardiomyopathy

The histologic alterations in dilated cardiomyopathy are nonspecific because they do not point to a specific etiology. Dilated cardiomyopathy occasionally exhibits similar clinical symptoms and histopathology to those of cardiac sarcoidosis. In particular, when typical granulomas are not formed in endomyocardial biopsy, it is occasionally difficult to distinguish between the interstitial phase of cardiac sarcoidosis and dilated cardiomyopathy. A fibrotic pattern is one of the histologic findings that can distinguish between both conditions. Fibrosis is pronounced in the myocardium with a mosaic pattern in cardiac sarcoidosis, whereas diffuse fibrosis in extensively injured cardiomyocytes is often seen in dilated cardiomyopathy [24,36].

Additionally, fatty infiltration can occur within loose connective tissue around granulomas in cardiac sarcoidosis, whereas there is no fatty infiltration within fibrous granulation tissues in dilated cardiomyopathy [24]. It is unclear whether fatty infiltration is the structural alteration resulting from granulomatous inflammation. Fatty infiltration may cause electrical disturbances and fatal dysrhythmias.

## 6. Conclusions

Sarcoidosis is a rare disease of isolated or diffuse granulomatous inflammation. Cardiac sarcoidosis is sometimes a fatal disorder, and it is important to accurately diagnose it to prevent sudden death due to fatal dysrhythmia. Although endomyocardial biopsy is invasive and has limited sensitivity for detecting granulomas, it is the only procedure that provides a definitive diagnosis of cardiac sarcoidosis. While classical histologic observations including noncaseating granulomas and multinuclear giant cells (typically Langhans type) are the most crucial, other findings such as microgranulomas, M1 macrophage accumulation, lymphangiogenesis, *C. acnes* components (detected by PAB antibody), confluent fibrosis, and fatty infiltration may be helpful to improve the sensitivity of endomyocardial biopsy. These novel histologic findings are based on the pathology of cardiac sarcoidosis.

Cardiac sarcoidosis is an involvement of a major organ and should be thoroughly studied and treated to avoid the risk of arrhythmia and of sudden death. Therefore, pathologists should carefully observe for granulomas and other histologic findings that indicate cardiac sarcoidosis, in order to achieve a timely and accurate diagnosis of the condition.

## Figures and Tables

**Table 1:** Diagnostic criteria for 2014 Heart Rhythm Society Expert Consensus Statement on the Diagnosis and Management of Arrhythmias Associated with Cardiac Sarcoidosis.

There are 2 pathways to a diagnosis of cardiac sarcoidosis: **1. Histological diagnosis from myocardial tissue** CS is diagnosed in the presence of non-caseating granuloma on histological examination of myocardial tissue with no alternative cause identified (including negative organismal stains if applicable). **2. Clinical diagnosis from invasive and non-invasive studies:** It is possible that there is CS if: (a) There is a histological diagnosis of extra-cardiac sarcoidosis *and* (b) One or more of following is present • Steroid +/− immunosuppressant responsive cardiomyopathy or heart block • Unexplained reduced LVEF (<40%) • Unexplained sustained (spontaneous or induced) VT • Mobitz type II 2nd degree heart block or 3rd degree heart block • Patchy uptake on dedicated cardiac PET (in a pattern consistent with CS) • Late Gadolinium Enhancement on CMR (in a pattern consistent with CS) • Positive gallium uptake (in a pattern consistent with CS) *and* (c) Other causes for the cardiac manifestation(s) have been reasonably excluded.

Abbreviations: CS, cardiac sarcoidosis; LVEF, left ventricular ejection fraction; VT, ventricular tachycardia; PET, positron emission tomography; CMR, cardiac magnetic resonance.

**Table 2:** The revised Japanese Ministry of Health and Welfare criteria for the diagnosis of cardiac sarcoidosis.

**a. Criteria for cardiac involvement** Cardiac findings should be assessed based on the major criteria and the minor criteria. Clinical findings that satisfy the following (1) or (2) strongly suggest the presence of cardiac involvement. (1) Two or more of the five major criteria (a) to (e) are satisfied (2) One in the five major criteria (a) to (e) and two or more of the three minor criteria (f) to (h) are satisfied. Major criteria (a) High-grade atrioventricular block (including complete atrioventricular block) or fatal ventricular arrhythmia (e.g., sustained ventricular tachycardia, and ventricular fibrillation) (b) Basal thinning of the ventricular septum or abnormal ventricular wall anatomy (ventricular aneurysm, thinning of the middle or upper ventricular septum, regional ventricular wall thickening) (c) Left ventricular contractile dysfunction (left ventricular ejection fraction less than 50%) or focal ventricular wall asynergy (d) ^67^Ga citrate scintigraphy or ^18^F-FDG PET reveals abnormally high tracer accumulation in the heart (e) Gadolinium-enhanced MRI reveals delayed contrast enhancement of the myocardium Minor criteria (f) Abnormal ECG findings: ventricular arrhythmias (non-sustained ventricular tachycardia, multifocal or frequent premature ventricular contractions), bundle branch block, axis deviation, or abnormal Q waves (g) Perfusion defects on myocardial perfusion scintigraphy (h)Endomyocardial biopsy: monocyte infiltration and moderate or severe myocardial interstitial fibrosis
**b. Diagnostic guidelines for cardiac sarcoidosis** (1) **Histological diagnosis group** (those with positive myocardial biopsy findings): cardiac sarcoidosis is diagnosed histologically when endomyocardial biopsy or surgical specimens demonstrate non-caseating epithelioid granulomas. (2) **Clinical diagnosis group** (those with negative myocardial biopsy findings or those not undergoing myocardial biopsy): the patient is clinically diagnosed as cardiac sarcoidosis (1) when epithelioid granulomas are found in organs other than the heart, and clinical findings strongly suggestive of the above-mentioned cardiac involvement are present; or (2) when the patient shows clinical findings strongly suggestive of pulmonary or ophthalmic sarcoidosis; at least two of the following five characteristic laboratory findings of sarcoidosis (bilateral hilar lymphadenopathy: high serum ACE activity or elevated serum lysozyme levels: high serum sIL-2R levels: significant tracer accumulation in ^67^Ga citrate scintigraphy or ^18^F-FDG PET: a high percentage of lymphocytes with a CD4/CD8 ratio of >3.5 in BAL fluid); and clinical findings strongly suggest the above-mentioned cardiac involvement.
**c. Diagnostic guidelines for isolated cardiac sarcoidosis** ** Prerequisite** 1. No clinical findings characteristic of sarcoidosis are observed in any organs other than the heart (The patient should be examined in detail for respiratory, ophthalmic, and skin involvements of sarcoidosis. When the patient is symptomatic, other aetiologies that can affect the corresponding organs must be ruled out.). 2. ^67^Ga scintigraphy or ^18^F-FDG PET reveals no abnormal tracer accumulation in any organs other than the heart. 3. A chest CT scan reveals no shadow along the lymphatic tracts in the lungs or no hilar and mediastinal lymphadenopathy (minor axis >10 mm). (1) **Histological diagnosis group**: Isolated cardiac sarcoidosis is diagnosed histologically when endomyocardial biopsy or surgical specimens demonstrate non-caseating epithelioid granulomas. (2) **Clinical diagnosis group**: Isolated cardiac sarcoidosis is diagnosed clinically when the criterion (d) and at least three other criteria of the major criteria (a) to (e) are satisfied. When the patient meets at least four criteria for cardiac involvement other than the criterion (d), or when the patient meets the criteria (b) and (d) plus one of the remaining criteria, the patient should be suspected to have isolated cardiac sarcoidosis.

Abbreviations: ^67^Ga, gallium-67; ^18^F-FDG PET, fluorine-18 fluorodeoxyglucose positron emission tomography; ACE, angiotensin converting enzyme; BAL, bronchoalveolar lavage; CT, computed tomography; ECG, electrocardiography; MRI, magnetic resonance imaging; sIL-2R, soluble interleukin 2 receptor.
